# Self-compassion and suicide risk: a moderated mediation model and evidence from Chinese universities

**DOI:** 10.3389/fpsyg.2023.1165723

**Published:** 2023-07-13

**Authors:** Dandan Ge

**Affiliations:** ^1^School of Education Science, Nanjing Normal University, Nanjing, Jiangsu, China; ^2^Nanjing Institute of Technology, Nanjing, Jiangsu, China

**Keywords:** self-compassion, suicide risk, negative emotions, resilience, college students

## Abstract

**Introduction:**

Suicide is a major social and public health problem in the world. It is important to identify protective and risk factors for suicide. This study aimed to investigate the relationship between self-compassion and suicide risk.

**Methods:**

1143 college students were surveyed by using Chinese Self-Compassion Scale (CSCS), Depression Anxiety Stress Scale-21 Chinese Version (DASS-21), Connor-Davidson Resilience Scale (CD-RISC), and Suicidal Behaviors Questionnaire-Revised (SBQ-R).

**Results:**

Negative self-compassion had a significant positive predictive effect on college students' suicide risk; in the model of negative self-compassion affecting suicide risk, negative emotions played a mediating role and the mediating role was moderated by resilience. Specifically, compared with low resilience, students with high resilience have a weaker ability to predict suicide risk by negative emotions.

**Discussion:**

Negative self-compassion is a risk factor for suicide risk, reducing negative self-compassion (self-judgment, isolation, and over-identification) and enhancing resilience has a guiding effect on suicide prevention and intervention.

## Introduction

Suicide is a major social and public health problem in the world, with about 804,000 people dying by suicide each year (O'Connor and Kirtley, 2018). In China, suicide is the leading cause of unnatural death in the age group from 15 to 34 years, and the suicide rate in the college student population is generally higher than in other populations and can even reach up to 2–4 times higher. Therefore, it is important to identify protective and risk factors with extensibility and develop targeted prevention and intervention measures to reduce the risk of suicide.

### Self-compassion affects suicide risk

Previous research on suicide risk has focused on environmental, emotional, and cognitive factors (Esposito et al., 2003; Liu et al., 2020). According to the integrated motivation-volition model of suicidal behavior (Garmezy et al., 1984; Joeng and Turner, 2015; O'Connor and Kirtley, 2018), self-attitude is an important influence on the formation of suicidal ideation and the development of suicidal behavior in individuals. However, research on suicide risk from the perspective of self-attitudes is still lacking (Liu et al., 2020). Self-compassion, as a self-attitude, may play a role in the motivational stage of suicidal behavior. Self-compassion refers to an individual's attitude toward himself or herself in the context of perceived failure, inadequacy, or distress. Self-compassion contains six components: self-kindness and self-judgment, common humanity and isolation, and mindfulness and over-identification (Neff et al., 2021). These different components combine to represent a self-compassion mindset. While most researchers in previous studies have examined self-compassion as a holistic construct, it has been argued that the inclusion of negative self-compassion (reverse scoring) into the self-compassion whole would exaggerate the inverse association of self-compassion with psychopathy (Montero-Marín et al., 2016), and thus, a two-factor model of self-compassion has been proposed. Self-kindness, common humanity, and mindfulness are categorized as positive self-compassion, while self-judgment, isolation, and over-identification are categorized as negative self-compassion (Muris et al., 2016). To go deeper, this study divided self-compassion into negative self-compassion and positive self-compassion to examine their relationship with suicide risk separately.

Positive self-compassion represents a positive psychological quality, which is considered to be an individual's protective coat against negative events. The higher the positive self-compassion score, the higher the individual's level of wellbeing, optimism, life satisfaction, and motivation, and the lower the depression, anxiety, stress, self-criticism, and physiological responses to coping with stress (Neff et al., 2007, 2021; Neff and Germer, 2013; Liu et al., 2020). The emotion regulation theory of self-compassion proposes that positive self-compassion alleviates negative emotions and can positively predict the development of an individual's mental health, whereas negative self-compassion creates problems with emotion regulation and negatively affects an individual's mental health (Finlay-Jones et al., 2017; Liu et al., 2022). Research has shown that positive self-compassion can reduce individual levels of suicidal ideation (Collett et al., 2016), whereas the relationship between negative self-compassion and suicide risk has received little attention and research. Previously, it was shown that negative self-compassion (self-judgment, isolation, and over-identification) were all significantly and positively associated with psychopathological traits (Muris et al., 2016). As the self-attitude associated with negative thought patterns, negative self-compassion can trap individuals in negative and distressing emotions (Joeng and Turner, 2015), increasing individual feelings of isolation and hopelessness (Rogers et al., 2017), while interpersonal theories of suicide point to individual feelings of belonging and burden as the most important factors in individual suicide production (O'Connor and Nock, 2014). Thus, the feelings of hopelessness and loneliness associated with negative self-compassion may enhance an individual's risk of suicide, but the mechanisms underlying negative self-compassion and suicide risk still need to be further explored. This leads to hypothesis 1: self-compassion significantly predicts suicide risk, while positive and negative self-compassion have different predictive effects.

### The mediating role of negative emotions

Negative emotions are a composite profile of an individual's subjective experience of unpleasant or distressing emotions. A meta-analysis showed that self-compassion was significantly related to negative emotions such as depression, anxiety, and stress (Neff, 2003). Positive self-compassion is an important potential protective factor for emotional problems such as depression and anxiety. Empirical studies have also found that positive self-compassion practices can substantially reduce negative emotions and enhance wellbeing, and the effects can be maintained for at least six months (Neff and Germer, 2013). A number of researchers have argued that the underlying psychological mechanism by which self-compassion exerts its efficacy is through emotion regulation strategies and that positive self-compassion reduces the use of maladaptive emotion regulation strategies of ruminative thinking, avoidance, and emotion suppression while promoting the use of adaptive emotion regulation strategies of acceptance and cognitive reappraisal, thus negative emotions are a mediating variable of interest (Finlay-Jones et al., 2017; Liu et al., 2020). On the other hand, both cross-sectional and longitudinal studies showed that negative emotions were significantly associated with suicide, and when the effects of some control factors were excluded the two were still significantly associated, suggesting that negative emotions are an important influencing factor in attempting suicide. This leads to hypothesis 2: negative emotions play a mediating role in the effect of self-compassion on suicide risk.

### The moderating role of resilience

Resilience is the ability of an individual to adapt well in the face of adversity or significant stress (Luthar et al., 2000; Yu and Zhang, 2007). As a positive psychological quality, it can buffer the effects of adverse environments on individual psychological development, minimize negative effects, and maximize positive growth and adaptation (Wang et al., 2021) and is often studied by foreign researchers as a protective factor corresponding to risk factors. The protective model of resilience assumes that risk factors can adversely affect individuals, but resilience may act as a moderator, and its interaction with risk factors can diminish the negative effects of risk factors and reduce the likelihood of negative consequences (Garmezy et al., 1984; Luthar et al., 2000). Rutter (2012) also proposed a similar perspective to describe the protective effect of resilience, where one variable can enhance the synergistic effect of another. Risk-protection mechanisms focus on protective factors to mitigate the negative effects of risk factors, while protection–protection mechanisms focus on the interaction of different protective factors to buffer possible negative effects, and this interaction may change with different levels of protective factors. The protective model of resilience has been validated by domestic and international studies, and the process of resilience is actually the process by which individuals mobilize their own protective and risk factor interactions (Kumpfer and Bluth, 2004). Based on this, this study proposed hypothesis 3: resilience plays a moderating role between self-compassion, negative emotions, and suicide risk.

**Figure 1:**
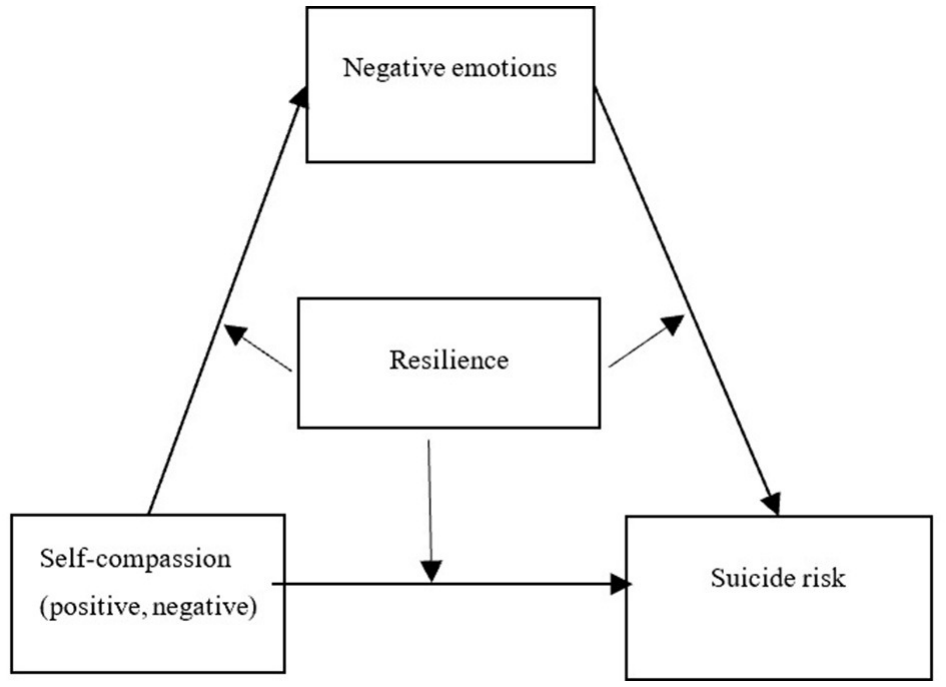
Analytical model.

The mechanisms by which positive and negative self-compassion affect suicide risk have rarely been explored separately from a dialectical perspective. In this study, we use the Integrated Motivation-Will Model of Suicidal Behavior, the Protective Model of Resilience, and the Emotion Regulation Theory of Self-compassion as the basis for a dialectical perspective. We construct a moderated mediation model (see Figure 1) to explore the influence of positive self-compassion and negative self-compassion on suicide risk and their internal mechanisms of action and try to answer the question of what (how) and under what conditions (when) self-compassion (negative and positive) affects suicide risk, to enrich the study of suicide mechanisms among college students and provide targeted suggestions and measures for suicide prevention and intervention among college students. The aim is to enrich the study of suicide mechanisms among college students and to provide targeted recommendations and measures for suicide prevention and intervention among college students.

## Materials and methods

### Participants and procedures

The whole-group sampling method was used to select college students in one part of Jiangsu Province to participate in this research study, and the group administration was conducted in the mental health class. Based on the voluntary participation and the completion of the questionnaire, 1,143 valid questionnaires were collected, among which 633 (55.4%) were male students and 510 (44.6%) were female students; 650 (56.87%) were students from urban areas, and 493 (43.13%) were rural students; 153 (13.39%) were students with left-behind experience and 990 students (86.61%) were not with left-behind experience; 389 students (34.03%) majoring in arts, 153 students (13.39%) majoring in science, and 601 students (52.58%) majoring in engineering; the average age of students was (18.91 ± 0.99) years. The demographic characteristics of the surveyed students are shown in Table 1.

**Table 1:** Frequency distribution of demographic characteristics of the sample (*N* = 1,143).

**Variables**	**Classification**	***N***	**%**
Gender	Male	633	55.38
	Female	510	44.62
Place of origin	Urban	650	56.87
	Rural	493	43.13
Left-behind experience	Yes	153	13.39
	No	990	86.61
Majors	Liberal art	389	34.03
	Science	153	13.39
	Engineering	601	52.58
Ages		18.91 (M)	0.99 (SD)

### Measurements

#### Depression anxiety stress scale-21 Chinese version

The Depression-Anxiety-Stress Self-Rating Scale was translated by Gong et al. (2010). The scale has 21 items and is scored on a four-point scale, with a higher total score indicating more severe negative emotions. The Cronbach alpha coefficient of the scale in this study was 0.96, and the results of the validation factor analysis were χ2/df = 5.42, CFI = 0. 956, TLI = 0. 942, and RMSEA = 0. 062, indicating that the internal consistency reliability and structural validity of the scale were good.

#### Connor-davidson resilience scale

The Chinese version of the Mental Toughness Scale was revised by Yu and Zhang (2007). The scale consists of three factors: toughness, optimism, and strength, with 25 items and a five-point scale. The Cronbach alpha coefficient of the scale in this study was 0.96, and the results of the validation factor analysis were χ2/df = 4.97, CFI = 0. 945, TLI = 0. 935, and RMSEA = 0. 059, indicating good reliability and structural validity.

#### Chinese self-compassion scale

The Chinese version of the Self-Compassion Scale was translated by Chen et al. (2011). The scale consists of six subscales: Self-kindness, Mindfulness, Common humanity, Isolation, Self-judgment and Over-identification with 26 questions and a five-point scale, with the first three dimensions constituting positive self-compassion and the last three dimensions constituting negative self-compassion. The Cronbach alpha coefficients of the subscales were 0.89 and 0.89, respectively, and the results of the validation factor analysis were χ2/df = 4.63, CFI = 0. 968, TLI = 0. 953, and RMSEA = 0. 056, indicating that the internal consistency reliability and structural validity of the scale were good.

#### Suicidal behaviors questionnaire-revised

The Suicidal behaviors Questionnaire was developed by Osman et al. (2001). The scale consists of four questions, including an assessment of past suicidal ideation plans and behaviors, suicidal ideation in the last year, the threat of suicide, and the likelihood of future suicide, with a total score of 3 to 18, with higher total scores indicating a higher risk of suicide. The Cronbach alpha coefficient of the scale in this study was 0.73, and the results of the validation factor analysis were χ2/df = 1.60, CFI = 0. 999, TLI = 0. 997, and RMSEA = 0. 023, indicating good internal consistency reliability and structural validity of the scale.

### Common method bias test

Data for this study were obtained from subjects' self-reports and may be subject to common method bias. It was controlled for by standardizing the guiding words for the administration process and adopting reverse scoring, while the Harman one-way test was used, and the results showed that 10 factors had a characteristic root greater than one, and the first factor explained 26.38% of the variance, which was less than the critical criterion of 40%, thus there was no serious common method bias.

## Results

### Descriptive statistics and correlations

To further understand the differences in demographic characteristics of the variables, independent sample *t*-tests were used to compare the variability of each variable among students of different genders, different places of origin, and whether they had left-behind experience, respectively. F-test was used to test the variability of each variable among students with different majors.

The results (Table 2) showed that there were significant gender differences in negative self-compassion and negative emotions among college students, which showed that male students had higher negative self-compassion and negative emotions than female students, and the differences were statistically significant with medium effect sizes. There were significant differences in negative emotions and resilience among college students in their places of origin, which showed that urban students had higher negative emotions and lower resilience than rural students, with statistically significant differences and small effect sizes. There were significant differences in negative self-compassion, negative emotion, resilience, and suicide risk among college students with and without left-behind experience. College students with left-behind experience have lower resilience than those without left-behind experience, while negative self-compassion, negative emotions, and suicide risk are higher than those without left-behind experience. There were significant differences in negative self-compassion and negative emotions among students with different majors. Multiple comparisons showed that negative self-compassion and negative emotions were lower among liberal arts students compared to engineering and science students.

**Table 2:** Test of mean, standard deviation, and significant differences of different demography characteristics (M ± SD).

**Demography indicators**		**N**	**PSC**	**NSC**	**NE**	**Resilience**	**Suicide risk**
Gender	Male	633	45.00 ± 3.12	38.54 ± 8.99	14.32 ± 12.73	67.85 ± 16.49	4.25 ± 2.14
	Female	510	45.62 ± 7.41	36.06 ± 8.69	10.1 ± 11.1	67.92 ± 15.04	4.22 ± 2.22
*t*			−1.34	4.71^***^	5.99^***^	−0.07	0.21
*Cohen's d*				0.28	0.36		
Place of origin	Urban	650	45.34 ± 7.70	37.52 ± 8.64	13.17 ± 12.77	67.03 ± 15.33	4.21 ± 2.11
	Rural	493	45.20 ± 7.80	37.32 ± 9.34	11.47 ± 11.35	69.00 ± 16.46	4.27 ± 2.26
*t*			0.30	0.38	2.36^*^	−2.09^*^	−0.51
*Cohen's d*					0.14	0.12	
Left-behind experience	Yes	153	45.37 ± 7.75	39.25 ± 8.90	16.22 ± 12.9	64.94 ± 15.39	4.78 ± 2.86
	No	990	45.27 ± 7.74	37.15 ± 8.92	11.85 ± 11.99	68.22 ± 15.88	4.15 ± 2.04
*t*			0.15	2.71^**^	4.15^***^	−2.53^**^	2.61^**^
*Cohen's d*				0.24	0.36	0.22	0.23
Majors	Science (1)	153	43.93 ± 7.02	38.33 ± 8.91	13.79 ± 13.66	66.92 ± 15.57	4.48 ± 2.65
	Engineering (2)	601	45.40 ± 7.86	38.18 ± 8.84	13.14 ± 12.41	67.54 ± 15.55	4.22 ± 2.03
	Liberal art (3)	389	45.62 ± 7.78	35.94 ± 8.93	10.82 ± 11.09	68.78 ± 16.40	4.15 ± 2.18
*F*			2.78	8.42^***^	5.41^**^	1.05	1.23
				(1)>(3);(2)>(3)	(1)>(3);(2)>(3)		

PSC, Positive self-compassion; NSC, Negative self-compassion; NE, Negative emotions; ^*^p < 0.05, ^**^p < 0.01, and ^***^p < 0.001.

Pearson's correlation analysis found that positive self-compassion among college students was significantly negatively related to both negative emotions and suicide risk and significantly positively related to resilience, while negative self-compassion was significantly positively related to negative emotions and suicide risk and significantly negatively related to resilience. Negative emotions were significantly negatively correlated with resilience and significantly positively correlated with suicide risk, and resilience was significantly negatively correlated with suicide risk, the results of which are presented in Table 3.

**Table 3:** Descriptive statistics and correlation analysis for each variable.

**Variables**	**1**	**2**	**3**	**4**	**5**	**6**	**7**	**8**	**9**	**10**	**11**
PSC	1										
Self-kindness	0.912^***^	1									
Common humanity	0.761^***^	0.526^***^	1								
Mindfulness	0.881^***^	0.739^***^	0.510^***^	1							
NSC	0.042	−0.137^***^	0.364^***^	−0.095^***^	1						
Self-judgment	−0.010	−0.108^***^	0.348^***^	−0.049	0.906^***^	1					
Isolation	−0.034	−0.141^***^	0.346^***^	−0.111^***^	0.900^***^	0.699^***^	1				
Over-identification	−0.045	−0.124^***^	0.288^**^	−0.091^***^	0.910^***^	0.739^***^	0.752^***^	1			
Negative emotions	−0.138^***^	−0.221^***^	0.081^***^	−0.229^***^	0.58^***^	0.489^***^	0.534^***^	0.530^***^	1		
Resilience	0.517^***^	0.512^***^	0.312^***^	0.462^***^	−0.181^***^	−0.075^*^	−0.225^***^	−0.203^***^	−0.343^***^	1	
Suicide risk	−0.121^***^	−0.196^***^	0.032	−0.127^***^	0.319^***^	0.274^***^	0.292^***^	0.304^***^	0.413^***^	−0.220^***^	1
M	45.28	17.56	13.02	14.70	37.44	14.08	11.56	11.79	12.44	67.88	4.24
SD	7.74	3.62	2.53	2.85	8.942	3.56	3.27	3.06	12.20	15.85	2.17
Cronbach α	0.89	0.80	0.69	0.78	0.89	0.74	0.77	0.73	0.96	0.96	0.73

PSC, positive self-compassion; NSC, negative self-compassion; ^*^p < 0.05, ^**^p < 0.01, and ^***^p < 0.001.

### A test of a mediated model with moderation

According to Wen and Ye (2014), all variables were standardized, demographic variables were controlled, and moderated mediating effects tests were conducted. Analyses were conducted using MODEL59 of the SPSS macro program PROCESS, with positive self-compassion and negative self-compassion as independent variables, suicide risk as a dependent variable, negative emotions as a mediating variable, and resilience as a moderating variable, respectively, with a bias-corrected percentile Bootstrap method test with 5000 replicate samples and 95% confidence intervals calculated. The parameters of each equation are shown in Tables 3, 4.

**Table 4:** Self-compassion and resilience predict negative emotions (equation 2).

**Predictor variables**	**β**	**SE**	***t***	**95% CI**
Positive self-compassion	0.05	0.03	1.48	[−0.02, 0.11]
Resilience	−0.36	0.03	−11.43^***^	[−0.43, −0.30]
Positive self-compassion^*^resilience	0.06	0.02	2.82^**^	[0.02, 0.10]
Negative self-compassion	0.51	0.02	21.43^***^	[0.47, 0.56]
Resilience	−0.24	0.02	−10.25^***^	[−0.29, −0.20]
Negative self-compassion^*^resilience	−0.02	0.02	−1.34	[−0.06, 0.01]

^*^p < 0.05, ^**^p < 0.01, *^***^p < 0.001.

Equation 1 tests whether the direct effect of self-compassion (positive self-compassion and negative self-compassion) on suicide risk is moderated by resilience. Table 5 shows that the negative prediction of suicide risk by positive self-compassion was not significant and the positive prediction of suicide risk by negative self-compassion was significant; the negative prediction of suicide risk by resilience was not significant, the effect of the interaction between positive empathy and resilience on suicide risk was not significant, and the effect of the interaction between negative compassion and resilience was not significant.

**Table 5:** Self-compassion, negative emotions, and resilience predict suicide risk (equation 1 and equation 3).

**Predictor variables**	**β**	**SE**	***t***	**95% CI**
Positive self-compassion	−0.04	0.03	−1.13	[−0.10, 0.03]
Resilience	−0.04	0.03	−1.15	[−0.10, 0.03]
Positive self-compassion^*^resilience	−0.03	0.02	−1.30	[−0.07, 0.01]
Negative emotions	0.39	0.03	13.34^***^	[0.33, 0.44]
Negative emotions^*^resilience	−0.12	0.02	−5.81^***^	[−0.16, −0.08]
Negative self-compassion	0.15	0.03	4.77^***^	[0.09, 0.22]
Resilience	−0.06	0.03	−2.07^*^	[−0.11, 0.00]
Negative self-compassion^*^resilience	−0.05	0.03	−1.77	[−0.09, 0.00]
Negative emotions	0.30	0.03	8.77^***^	[0.23, 0.36]
Negative emotions^*^resilience	−0.10	0.03	−3.98^***^	[−0.15, −0.05]

^*^p < 0.05, ^**^p < 0.01, *^***^p < 0.001.

Equation 2 tests whether self-compassion (positive self-compassion and negative self-compassion) on negative emotions is moderated by resilience. As seen in Table 4, positive self-compassion was not a significant predictor of negative emotions; negative self-compassion was a significant positive predictor of negative emotions; resilience was a significant negative predictor of negative emotions; the interaction between positive self-compassion and resilience was significant, but the interaction between negative self-compassion and resilience was not significant.

Equation 3 tests whether negative emotions and suicide risk are directly moderated by resilience. Table 5 shows that negative emotions significantly and positively predict suicide risk; the interaction between negative emotions and resilience has a significant effect on suicide risk. Thus, the second half of the mediated model's path was moderated by resilience.

**Figure 2:**
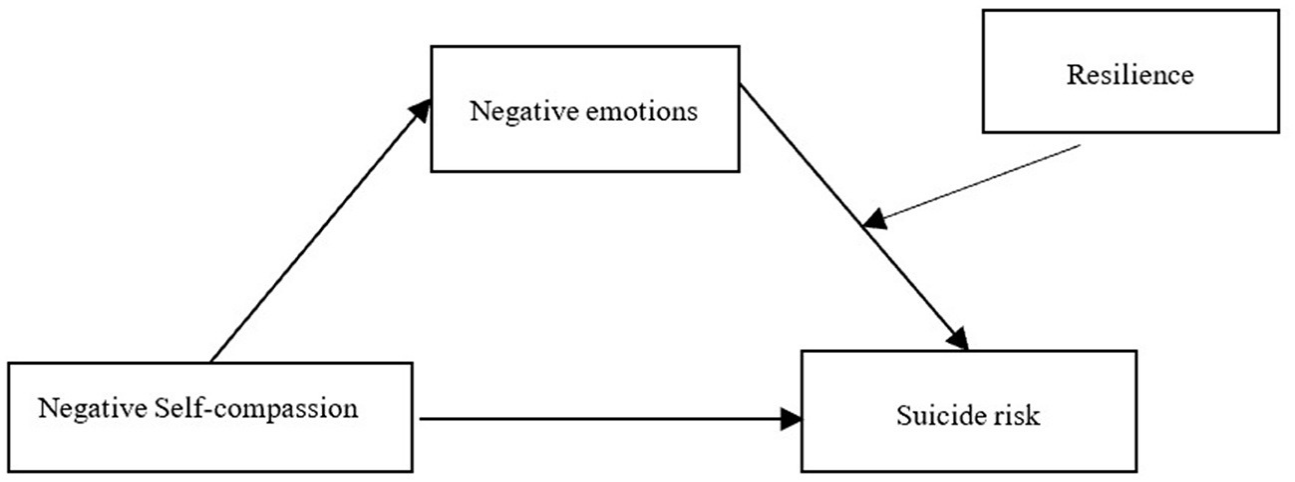
Post-validation model.

As shown in Tables 4, 5, the mediated model with moderation was only present in the mediated model of negative self-compassion, negative emotions, and suicide risk. Further analysis revealed: The low resilience group had a direct effect value of 0.20, 95% CI [0.11, 0.28], and a mediating effect of 0.21, 95% CI [0.14, 0.29]; the high resilience group had a direct effect value of 0.11, 95% CI [0.03, 0.18] and a mediating effect of 0.10, 95% CI [0.05, 0.14]. However, whether the mediating effect with moderation really exists or not, INDEXES has to be judged. The results showed that in the mediating model of negative self-compassion, negative emotions affecting suicide risk, INDEX = −0.07, 95% CI [−0.10, −0.03] does not contain 0, indicating that the mediating effect with moderation is significant. Among negative self-compassion, negative emotions (mediator) and suicide risk, the mediator of negative emotions was moderated by resilience (Figure 2).

**Figure 3:**
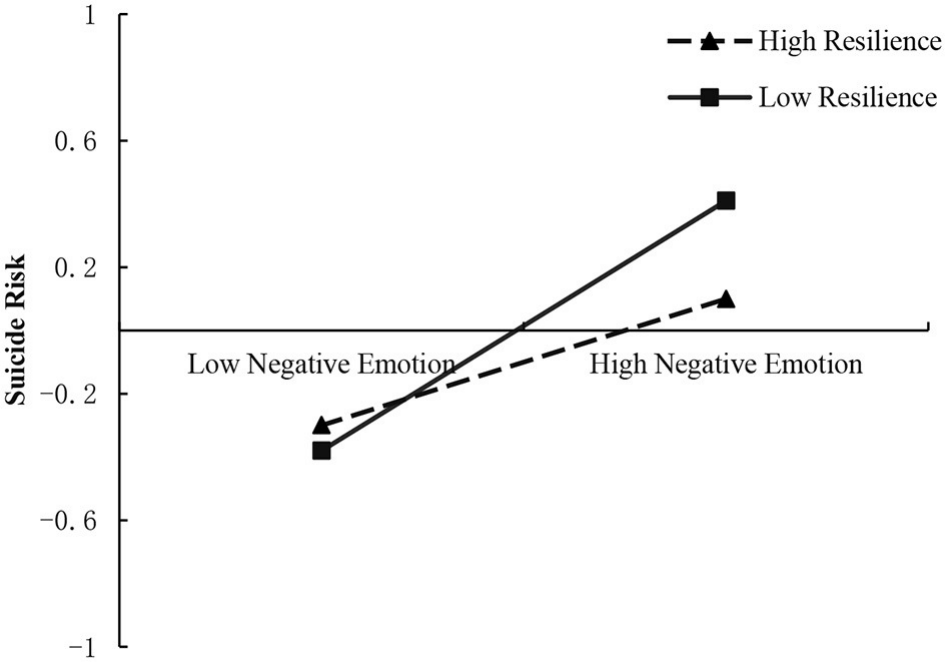
Moderating role of resilience between negative emotions and suicide risk.

To more clearly explain the moderating effect of resilience, a simple slope analysis test was conducted by dividing resilience into two groups: low group (M−1SD) and high group (M+1SD), and as shown in the Figure 3, the positive predictive effect of negative emotions on suicide risk was significant when resilience levels were low (bsimple = 0.40, *t* = 9.86, *p*<*0*.001); at higher levels of resilience, the negative emotions had a weaker predictive effect on suicide risk although they also had a positive predictive effect (bsimple = 0.20, *t* = 4.51, *p* < 0.01), indicating a decreasing trend in the predictive effect of negative emotions on suicide risk as individuals' level of resilience increased.

## Discussion

### Analysis of self-compassion, negative emotions, resilience, and suicide risk demographic variables

The study found that there were individual differences in self-compassion, negative emotions, resilience, and suicide risk. Firstly, the gender characteristics showed that male students had significantly higher negative self-compassion and negative emotions than female students. According to social role theory, socialization processes and gender roles may have an impact on college students' social adjustment (Eagly, 2009). Female students are more interpersonally oriented and receive more support from their peers than male students. In addition, there are differences in the way individuals of different genders regulate bad emotions, mainly in that male students are more inclined to adopt aggressive and repressive behaviors to vent their bad emotions, while female students have better understanding and tolerance; therefore, female students have lower negative self-compassion and negative emotions. Secondly, most humanity majors pay more attention to humanistic care and humanistic cultivation, which reduces negative self-compassion to some extent. Meanwhile, compared with engineering and science students, liberal arts students may be better at paying attention to expressing and regulating their emotions, have richer and more effective emotion regulation strategies, and thus have fewer negative emotions. Once again, there is variability in the levels of resilience and negative emotions by the place of origin, as evidenced by the fact that rural students have higher levels of resilience and fewer negative emotions. Rural students have higher levels of control and management skills than their urban counterparts and are more capable of resisting stress, overcoming adversity, and dealing with crises, which facilitate their ability to actively mobilize resources around them and solve problems. Finally, students with the left-behind experience had significantly higher levels of negative self-compassion, negative emotions, and suicide risk and lower levels of resilience than those without the left-behind experience. This study validates the damaging effect of the left-behind experience on individual mental health, where adverse experiences associated with parental absence in early childhood can lead to individual emotional and behavioral problems (Tang et al., 2018; Zhao et al., 2019). On the one hand, with left-behind experience, students need to cope with personal and academic difficulties alone, and thus, they are more likely to experience psychopathological symptoms such as anxiety and depression, thus increasing the risk of suicide. On the other hand, parental absence may bring a sense of abandonment, despair, and loneliness that gradually transform into low self-esteem, which, combined with the higher level of closed cognition and dogmatic thinking formed at an early age, makes students with left-behind experiences more prone to self-doubt and self-denial when faced with the impact of negative events and exhibit having a lower sense of personal value and meaningfulness of life.

### The mediating role of negative emotions

This study found that negative self-compassion had a significant positive predictive effect on suicide risk, and more importantly, it also found that negative emotions mediated the effect of negative self-compassion on suicide risk, i.e., negative self-compassion had an indirect effect on suicide risk through negative emotions, validating hypotheses 1 and 2. Previous studies have also shown that an increase in positive self-compassion is more directly associated with positive psychological states while an increase in negative self-compassion is more directly associated with an increase in negative emotions such as depression, stress, and anxiety (Neff, 2016). The same validates the emotion regulation theory of self-compassion (Finlay-Jones et al., 2017; Liu et al., 2020). Emotion regulation strategies are the psychological mechanisms by which self-compassion is effective, individuals with negative self-compassion are more inclined to adopt negative coping styles of avoidance and expression inhibition, and individuals are prone to problems with emotion regulation, exacerbating the negative emotions experienced by individuals (Liu et al., 2020). Cognitive models also highlight that negative self-compassion amplifies the feelings of helplessness and despair experienced by individuals, sees themselves as the center of suffering, and perpetuates their immersion in painful emotions, thus exacerbating negative emotions and increasing the risk of suicide (Joeng and Turner, 2015; Liu et al., 2020). Therefore, negative self-compassion among college students can positively predict suicide risk through the mediating effect of negative emotions. If students have higher levels of negative self-compassion, they are more likely to experience negative emotions, and once negative emotions accumulate, the likelihood of suicide risk emergence increases.

### The moderating role of resilience

This study found that resilience moderates the “relationship between negative emotions and suicide risk” pathway, testing hypothesis 3. It supports the protective model of resilience (Garmezy et al., 1984; Luthar et al., 2000; Rutter, 2012), which suggests that resilience can buffer or compensate for the effects of risk factors and reduce the negative effects of risk factors on individuals. Although negative emotions, as a factor detrimental to an individual's mental health, positively predict suicide risk among college students, with higher negative emotions associated with greater suicide risk, as a positive protective factor, an individual's own resilience can quickly mobilize an individual's own resources to cope with negative emotions and can be triggered when negative emotions are pent up, effectively mitigating the negative effects of negative emotions. Kumpfer and Bluth's (2004) resilience framework theory also emphasizes that after experiencing a negative event, individuals can mitigate the impact of the negative event through positive cognition and good emotional control to promote positive evaluations and feelings and reduce the adverse effects of risk factors on themselves. Specifically, the risk of suicide increases with the accumulation of negative emotions; however, in the presence of highly perceived negative emotions, individuals with high levels of resilience use a wealth of internal and external resources to regulate negative emotions and attenuate the negative effects of negative emotions on suicide risk. At the same time, this study further investigated the changes in negative emotions and suicide risk at different levels of resilience, showing that the moderating effect of resilience on the relationship between negative emotions and suicide risk did not show a qualitative change in the relationship but only a quantitative change. Regardless of the level of resilience, an increase in negative emotions brought about an increase in suicide risk, but there was indeed a significant tendency for the impact of negative emotions on suicide risk to slow down as the level of resilience increased. This result may be due to the higher level of positive emotions in high-resilience individuals, who can expand their internal cognitive, attentional, and behavioral resources and can recognize and regulate their emotions well during adversity, as well as studies show that high-resilience individuals are more sensitive to emotional reactions and recover more quickly from negative emotional arousal than low resilience individuals (Xi et al., 2015). Individuals with low resilience lack the ability to regulate their emotions flexibly and effectively and have difficulty dealing with their negative emotions in a timely manner, thus exacerbating the negative effect of negative emotions on suicide risk. Compared to individuals with low resilience levels, those with high resilience have stronger immunity, experience less depression and anxiety, and have better behavioral performance. Thus, high levels of resilience are more positive and effective in protecting against suicide risk when college students are faced with more negative emotions.

## Conclusion

From this study, it was concluded that negative self-compassion significantly and positively predicted suicide risk among college students, whereas positive self-compassion did not have a significant predictive effect. Negative emotions partially mediated the effect of negative self-compassion on suicide risk, and negative self-compassion enhanced individuals' suicide risk through negative emotions. The moderated mediation model of negative self-compassion affecting suicide risk among college students holds true, in which, resilience moderates the relationship between negative emotions and suicide risk, and high levels of resilience buffer the effect of negative emotions on suicide risk.

On the one hand, the study has some reference significance for systematically understanding the mechanism of action of the relationship between self-compassion and suicide risk; on the other hand, examining the mediating and moderating roles of negative emotions and resilience in self-compassion and suicide risk has the following implications for the prevention and intervention of suicide risk among college students: firstly, individual students should reduce and change the individual's negative self-compassion, such as self-judgment, sense of isolation, and over-identification, which to some extent helps to mitigate the risk of suicide. Secondly, schools should strengthen the relevance of resilience cultivation for college students to enhance individuals' ability to cope with risk, so that individuals can adjust their physical and mental health in the face of adversity and setbacks to achieve the goal of reducing suicide risk. Finally, schools should give more attention to college students with left-behind experiences as they are prone to induce negative emotions, damage their psychological health, and develop more suicide risks due to the absence of parents and lack of care during childhood, and their psychological needs cannot be met; schools should help them reconstruct their kinship alliance as a way to compensate for the lack of family warmth.

## Data availability statement

The raw data supporting the conclusions of this article will be made available by the authors, without undue reservation.

## Ethics statement

The studies involving human participants were reviewed and approved by the Mental Health Center of Nanjing Institute of Technology. The patients/participants provided their written informed consent to participate in this study.

## Author contributions

DG conducted all the research and wrote this paper.
